# Bilateral reversible optic neuropathy as the first sign of Waldenström macroglobulinema

**DOI:** 10.3205/oc000240

**Published:** 2024-06-25

**Authors:** Yoshiaki Shimada, Yoshiki Akatsuka, Kazuya Nokura

**Affiliations:** 1 Department of Ophthalmology, Fujita Health University School of Medicine, Aichi, Japan; 2 Department of Hematology and Oncology, Fujita Health University School of Medicine, Aichi, Japan; 3 Department of Neurology, Daiyukai General Hospital, Aichi, Japan

**Keywords:** monoclonal gammopathy, multiple myeloma, optic neuropathy, Waldenström macroglobulinemia

## Abstract

**Objective:**

To report a case of bilateral reversible optic neuropathy as the first sign of Waldenström macroglobulinemia (WM).

**Methods:**

Observational case report.

**Results:**

A 52-year-old man had a sudden loss of vision in the left eye. Examinations revealed the presence of a serum monoclonal immunoglobulin (IgM kappa) in the serum. Even after a session of steroid pulse therapy, optic neuropathy became bilateral and then resolved almost completely after 4 months. The condition progressed to WM with multiorgan lesions years later. There was no evidence of optic neuropathy recurrence. The literature revealed two cases of monoclonal gammopathy (MG): a 64-year-old man with multiple myeloma (MM) with IgA lambda and a 51-year-old man with MM with IgG kappa. These cases have similar conditions: 1) visual reduction as an initial symptom of MG, 2) bilateral involvement, 3) no sign of central nervous system (CNS) infiltration shown by normal brain magnetic resonance images, and 4) recovery to a visual acuity of ≥1.0 bilaterally with no reoccurrence. The excessive Igs or B-cell hyperactivity may activate an autoimmune mechanism that reversibly interferes with the bilateral optic nerves.

**Conclusion:**

Bilateral optic neuropathy was the initial symptom of WM. There was no evidence of CNS infiltration; it recovered and then did not reoccur. The pathogenesis remained unknown, but two cases of MG were reported in the literature with remarkably similar conditions.

## Introduction

Waldenström macroglobulinemia (WM) is a distinct hematologic malignancy characterized by lymphoplasmacytic bone marrow infiltration and the presence of immunoglobulin (Ig) M monoclonal protein [1]. Ocular manifestations are usually caused by hyperviscosity syndrome due to elevated serum IgM levels [1]; however, studies have reported that optic neuropathy unrelated to hyperviscosity syndrome is associated with WM [2], [3], [4], [5], [6], [7], [8], [9], sometimes as an initial symptom [4], [5], [8]. Optic neuropathy is caused by central nervous system (CNS) infiltration in some WM cases, which has been referred to as the Bing-Neel syndrome [2], [3], [5], [6], [7], [8], [9]. Additionally, visual [2], [3], [8] and life [5] prognoses have often been poor despite extensive therapies. Here, we report a case of WM that began with a sudden loss of light perception in the left eye. Optic neuropathy was initially substantially bilateral, then became distinctly bilateral, and finally resolved. There was no other symptom or lesion, although a serum monoclonal Ig (IgM kappa) was detected. The case remained as monoclonal gammopathy of undetermined significance (MGUS) [10]. The patient developed multiorgan lesions, which were diagnosed as WM years later. There was no evidence of visual reduction recurrence.

## Case description

**Figure 1:**
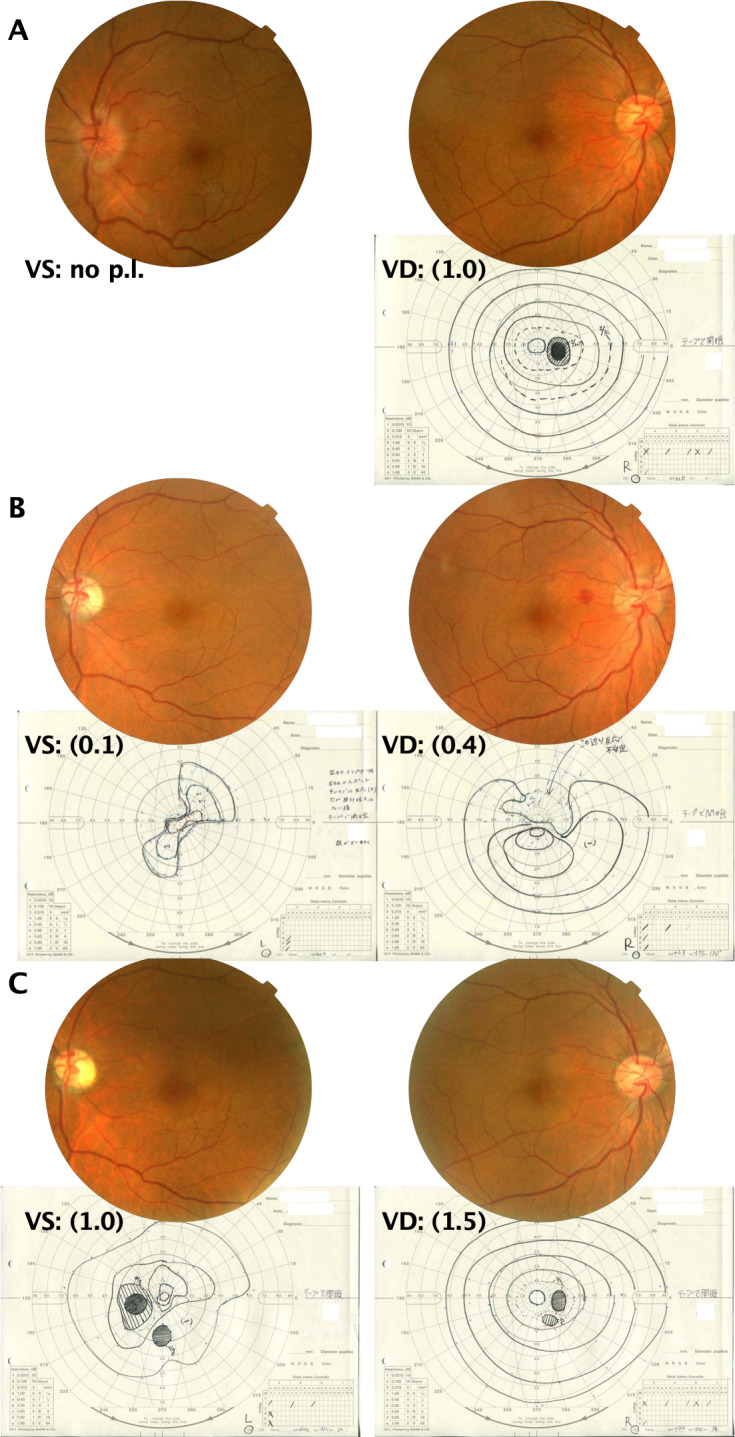
Fundus images and Goldmann visual field testing A: At the first visit. Fundus images revealed edema on the disc of the left eye. Retinal veins were slightly dilated in the left eye, but with no string-of-sausage segmentation. Goldmann visual field testing was nonrecordable in the left eye. There was a slight enlargement of the blind spot in the right eye. B: After 2 weeks. Fundus images revealed a resolved edema on the disc in the left eye. A small subretinal hemorrhage could be observed between the optic disc and the fovea in the right eye. Goldmann visual field testing revealed a slightly recovered left eye, but the right eye developed a paracentral scotoma. C: After 4 months. Fundus images revealed ophthalmoscopically apparent optic atrophy in the left eye. Goldmann visual field testing revealed significantly recovered bilateral eyes.

A 52-year-old man was unnerved after having a sudden loss of vision in the left eye. He had no other symptoms, not even eye pain, and his medical and family histories were unremarkable. The patient was seen with a severe optic neuropathy of the left eye (no light perception and optic disc swelling). The visual acuity (VA) was 1.0 in the right eye, and the left eye showed no light perception and had a relative afferent pupillary defect. The optic disc was swollen, and retinal veins were slightly dilated in the left eye, but with no vascular changes characteristic of hyperviscosity syndrome, such as venous congestion with sausage-like segmentation (Figure 1A (Fig. 1)). The right eye had initially good vision (vessel density (VD), 1.0) and always showed a normal optic disc. 

**Figure 2:**
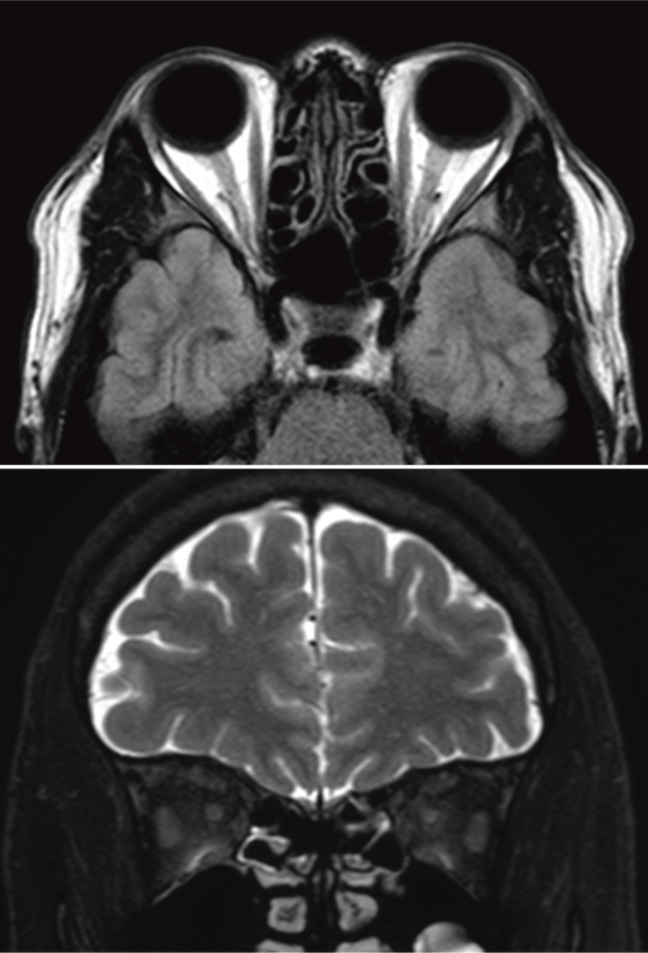
Brain and orbital magnetic resonance images obtained urgently during the initial visit

Brain and orbital non-contrast enhanced magnetic resonance imaging (MRI) was immediately obtained during the initial visit and did not reveal abnormal findings (Figure 2 (Fig. 2)). It was unfortunate that contrast-enhanced magnetic resonance imaging (MRI) could not be performed. If contrast had been performed, a left-right difference in the left and right optic nerve signals might have been noted.

The patient was hospitalized and received steroid pulse therapy, i.e., intravenous methylprednisolone at 1 g for 3 days, once. Laboratory tests revealed the following (value, normal range): slight anemia (13.2 g/dL, 13.3–16.9 g/dL), elevated segmented leukocyte level (74%, 38%–58%), low lymphocyte count (19%, 36%–47%) with normal white blood cell count (8,500/µL, 4,000–9,400/µL), elevated C-reactive protein level (3.8 mg/dL, <0.3 mg/dL), and high IgM level (1,524 mg/dL, 35–220 mg/dL). Autoantibodies were examined for anti-aquaporin 4 antibody, anti-myelin-associated-glycoprotein (MAG) autoantibody, antinuclear antibody, and anti-DNA antibody; all were negative.

After 2 weeks VA deteriorated even in the right eye (VD, 0.4) and a hemorrhage was seen between fovea and disc. There may of course be a delay between the eyes in the development of visual loss. The left eye had slightly recovered (visually significant (VS), 0.1) (Figure 1B (Fig. 1)). There was no eye pain throughout the course. 

A serum monoclonal Ig (IgM kappa) was detected, and monoclonal gammopathy (MG) was confirmed; however, the IgM level was not extremely high, with no other symptom/organ lesion. The patient was diagnosed with MGUS. VA recovered to 1.5 in the right eye and 1.0 in the left eye after 4 months of follow-up (Figure 1C (Fig. 1)). Chemotherapy was still withheld, and no treatment was provided, except steroid pulse therapy only once. However, the IgM level continuously increased after 3 years, and the patient developed anemia. The diagnosis of WM was made with kidney, spleen, or lymph node enlargements, and chemotherapy was started. There was no evidence of optic neuropathy recurrence.

## Discussion

Although Bing-Neel syndrome is believed to be extremely rare, optic neuropathy associated with WM can be due to this syndrome, i.e., the CNS infiltration in WM [1], [2], [3], [4], [5], [6], [7], [8], [9]. Anticancer treatment – chemotherapy – occasionally stabilizes optic neuropathy [4], [6], [7], [9]; however, visual [2], [3], [8] and life [5] prognoses are frequently poor. Blood disorders can also trigger ischemic optic neuropathy, but the visual prognosis is also poor for both arteritic and nonarteritic conditions [10].

In contrast, the patient herein had a loss of vision as an initial and very early symptom and bilateral optic neuropathy without any other signs of CNS involvement; he achieved almost complete recovery of vision, while MGUS [11] remained before progressing to WM.

Reversible acute loss of vision is also a characteristic of optic neuritis [12]. However, based on recently proposed diagnostic criteria [12], the patient reported here did not qualify as having either definite optic neuritis or possible optic neuritis due to the presence of painless, bilateral involvement, and no intrinsic signal on MRI.

The literature reports two MG cases, a 64-year-old man with multiple myeloma (MM) with IgA lambda of 1,060 mg/dL at onset [13] and a 51-year-old man with MM with IgG kappa of 3,020 mg/dL at onset [14] with remarkably similar conditions. Polyneuropathy-organomegaly-endocrinopathy-M protein-skin changes (POEMS) syndrome was excluded in the latter case [14]. The present case is the third report on bilateral reversible optic neuropathy with MG. These three cases have certain conditions in common: 1) visual reduction as an initial symptom of MG, 2) bilateral involvement, 3) no sign of CNS infiltration shown by normal brain MRI, and 4) recovered to VA of ≥1.0 bilaterally with [13] and without [14] chemotherapy and with no recurrence.

High Ig levels have been associated with the pathogenesis of reversible optic neuropathy, specifically, IgA binds to the nerve, [13] and IgG stimulates the infiltration of neutrophils into the nerve [13]. MG has been recently reported to be accompanied by autoimmune diseases [15], [16]. Patients with neuromyelitis optica, Kikuchi disease, Sjogren’s syndrome, and ankylosing spondylitis developed MGUS either coincidently or up to 10 years after being diagnosed with autoimmune conditions. B-cell hyperactivity is suggested as a hallmark of these autoimmune diseases, and immune-related conditions can be associated with an elevated MGUS and MM risk [15], [16]. The three cases of optic neuropathy as an initial symptom of MG have neither proven distinctive autoimmune disease nor autoantibodies; however, a related autoimmune mechanism may reversibly interfere with bilateral optic nerves.

## Conclusion

Bilateral optic neuropathy was the initial symptom of WM. There was no evidence of CNS infiltration; it recovered and then did not reoccur. The pathogenesis remained unknown, but the literature reported two MG cases with remarkably similar conditions.

## Notes

### Competing interests

The authors declare that they have no competing interests.
